# With a little help from our pediatrician: An intervention to promote mathematics-related home activities through regular well-child visits

**DOI:** 10.3389/fpsyg.2022.1051822

**Published:** 2022-12-05

**Authors:** Carlo Tomasetto, Jo-Anne LeFevre, Maria Chiara Passolunghi, Chiara De Vita, Veronica Guardabassi, Antonella Brunelli, Francesco Ciotti, Giancarlo Biasini

**Affiliations:** ^1^Department of Psychology Renzo Canestrari, University of Bologna, Bologna, Italy; ^2^Department of Psychology, Institute of Cognitive Science, Carleton University, Ottawa, ON, Canada; ^3^Department of Life Sciences, University of Trieste, Trieste, Italy; ^4^Azienda Unità Sanitaria Locale (AUSL) della Romagna, Cesena, Italy; ^5^Associazione Culturale Pediatri – Romagna (ACPR), Cesena, Italy; ^6^Private Practitioner, Cesena, Italy

**Keywords:** home mathematics environment, early numeracy, preschool, parent–child activities, intervention, pediatricians

## Abstract

**Introduction:**

Children’s involvement in mathematics-related activities in the home environment is associated with the development of their early numeracy over the preschool years. Intervention studies to promote parents’ awareness and provision of mathematics-related home activities are however scant. In this study we developed and tested the effectiveness of a non-intensive intervention program delivered by community pediatricians to promote mathematics-related activities in the home environment.

**Methods:**

Parents of 204 Italian children were invited to report on the frequency of mathematics-related home activities when children attended the first preschool year (3 years, 8 months of age on average) and, subsequently, the third preschool year (5 years, 6 months of age on average). At both waves, children were also assessed on their early numeracy. In occasion of the routine well-child visit at age 5, parents who were randomly allocated to the intervention condition (vs. a business-as-usual control condition) received guidance on age-appropriate home mathematics-related practices to sustain children’s numerical development.

**Results:**

Results revealed that parents in the intervention group improved their provision of home mathematics-related activities at the post-intervention assessment (relative to baseline) to a greater extent than parents in the control condition. No effect was observed on children’s early numeracy.

**Discussion:**

Overall, results are promising in suggesting that community pediatricians may be a resource to promote home mathematics-related activities though non-intensive low-cost interventions.

## Introduction

People need to build up solid competencies in mathematics to cope with a host of challenges in everyday life. Beyond undermining future academic achievement (Geary, 2011) and employment opportunities (Gross et al., 2009; Martin, 2018), shortage of mathematical skills also prevents people from using math knowledge and procedures to solve basic daily life problems (Jansen et al., 2016). Adults with poor mathematical skills, for example, may struggle with numerical information implied in health risks comprehension (Rolison et al., 2020) and basic medical practices, such as calculation of dose and timing of drug self-administration (Moore et al., 2011), with evident risks to health (Reyna et al., 2009; Peters, 2012). Efforts to improve mathematical competencies from the earliest life years should therefore be seen as a goal not only for the educational systems, but also for professionals and services involved in the promotion of individuals’ health and wellbeing at large. In this work, we evaluated the impact of a non-intensive intervention delivered by community pediatricians in the context of ordinary well-child visits. The goal of the intervention was to increase the frequency of mathematics-related activities in the home environment among parents of preschool-aged children, and to promote children’s early numeracy in the preschool years.

### The development of early mathematical skills

Mathematical competencies emerge early in life and undergo substantial development before children encounter formal teaching at school. Several milestones of numerical knowledge are typically acquired between age 2 and 6, such as the number-word sequence, the ability to map numerical symbols onto related quantities, the cardinality and ordinality principles, and basic arithmetical skills (e.g., simple additions and subtractions; LeFevre et al., 2010a; Siegler and Braithwaite, 2017; Litkowski et al., 2020). Numerical competencies acquired in the preschool years are among the strongest predictors of later academic achievement throughout primary and secondary school (Duncan et al., 2007; Watts et al., 2014; Geary et al., 2018; Davis-Kean et al., 2021).

Before attending school, the family environment is a crucial context in which children learn and practice their emerging mathematical competencies. Shared mathematics-related activities in the home – commonly referred to as “home numeracy” (Skwarchuk et al., 2014) – include direct numerical teaching, such as helping children practice counting or retrieving simple sums, and indirect experiences, such as when parents use numbers in their conversations with children, play number games, use measurement and numerals during cooking activities, and read storybooks with numerical content. Although it is plausible that children with greater numerical skills may elicit more number-related activities from their parents, longitudinal studies suggest that the frequency of home mathematics-related activities (Susperreguy et al., 2021; Authors, submitted) and the use of numerical language in parent–child conversations (Gibson et al., 2020) prospectively predict the growth of preschoolers’ numerical competencies over time, even after controlling for children’s numerical skills at baseline (see Mutaf Yıldız et al., 2020, for a review).

Provision of home numeracy activities is nonetheless highly variable across families, and not all children have equal opportunity to receive adequate support for their early numeracy development. Socio-demographic factors (e.g., parents’ instruction, child’s gender; see Saxe et al., 1987; Vandermaas-Peeler et al., 2012b), as well as beliefs and attitudes toward math may shape parents’ engagement in number-related activities (LeFevre et al., 2010b; Skwarchuk et al., 2014). Parents with positive attitudes toward mathematics tend to attribute more importance to math achievement (i.e., valuing of math; Eccles and Wigfield, 2002) and report more frequent engagement with home numeracy (Del Río et al., 2017; Susperreguy et al., 2018). This may be of special concern in countries, such as Italy, in which attitudes toward science, technology, and the STEMs in general are generally less favorable at the population level (European Commission, 2021), and the reported frequency for use of numerical skills and engagement in numeracy practices in everyday life is lower than in other industrialized countries (Jonas, 2018). Attempts should therefore be made to improve parental knowledge and attitudes toward early mathematics-related home activities, and support parents in providing richer home numeracy environments for their children (Niklas et al., 2016; Purpura et al., 2019).

### Interventions to promote mathematics-related home activities

Despite the spread of research on home numeracy over the last decade (Mutaf Yıldız et al., 2020), interventions to promote number-related practices in the home environment are still rare. In a meta-analysis of home-based interventions to improve literacy and numeracy outcomes among preschool-aged children, only 10 studies focused on mathematics-related outcomes were retained, as compared to 28 studies focused on literacy (Cahoon et al., 2022). Evidence however exists that the frequency of number-related activities and games, as well as the use of numerals in daily conversations, can be successfully improved. Increased involvement in shared mathematics-related activities with parents and higher mathematical skills were observed among children whose parents received structured, intensive programs with repeated sessions of information, guided play with children, and instruction on mathematics-related activities to be conducted at home (Starkey and Klein, 2000; Niklas et al., 2016; Dulay et al., 2019). Leyva et al. (2018), for example, invited parents of kindergartners from low-income Latino backgrounds to take part in an intensive 4-week training program in which participants were instructed to incorporate mathematical strategies (e.g., counting, matching quantities with numerical symbols) into daily cooking routines. Results revealed that children of parents who participated in the intervention showed improved numeracy skills. The intervention was especially effective for children who had lower numerical competencies at baseline, thus supporting the idea that parents can be effectively encouraged to include more mathematics-related activities in their children’s home environment.

Other studies showed that even non-intensive interventions, in which parents are provided with only minimal instruction, may also be effective. In a study with parents of preschoolers (Vandermaas-Peeler et al., 2012a), parent–child dyads were observed during a board game play session. In addition to the board game, half of the parents were given a list of suggested numeracy activities to incorporate into the game at their own discretion, but with no further instruction on how and when to do that. Results revealed that parents in the intervention condition not only performed more numerical activities, as prompted by the experimenter’s suggestions, but also provided more feedback on children’s number-related responses. In turn, children’s mathematics achievement improved following the intervention. Similarly, parents of four-year-old children increased their mathematics-related support when they were invited to incorporate number-related talk and activities (e.g., counting, comparing quantities, or doing basic operations) into ordinary cooking activities at home, without receiving any further specific training (Vandermaas-Peeler et al., 2012b).

In some cases, non-intensive interventions were effective in fostering mathematics-related activities in ordinary contexts outside the household, such as visits at museum exhibits (Vandermaas-Peeler et al., 2016; Braham et al., 2018) or shopping (e.g., Hanner et al., 2019). For example, Hanner et al. (2019) placed signs in grocery stores encouraging parents to interact with their children and pose them questions. In a numerical intervention condition, signs invited parents to engage in number-related talk (e.g., “Try asking … How many eggs are in a cartoon”?). In an active control condition, signs simply prompted parents to pose generic questions (e.g., “Try asking … What animal lays eggs?”), whereas in a neutral control condition no tip was provided. Observations of parents’ interactions with children revealed that in the numerical intervention condition, number-related talk was twice as frequent as in both the control conditions.

Overall, these findings suggest that even non-intensive interventions may be sufficient to raise parental awareness of numerous opportunities to include mathematics-related practice in their daily interactions with preschool-aged children.

### Promoting home numeracy through community pediatricians

In most industrialized countries, children and their parents access primary health consultation and pediatric check-ups on a regular basis, especially in the preschool years (Larson et al., 2016). In Italy, primary and preventive pediatric care – including routine well-child visits – is provided free of charge by the National Health System and parents are generally highly satisfied with the community pediatricians as the primary child health care providers (Corsello et al., 2016). Scheduled well-child visits thus provide community pediatricians with a unique opportunity to inform parents about a variety of issues pertaining to healthy child development.

Current guidelines for children’s primary healthcare already prompt pediatricians to carry out periodic screenings and provide guidance for parents on language acquisition (Council on Children With Disabilities et al., 2006; Committee on Practice and Ambulatory Medicine and Bright Futures Periodicity Schedule Workgroup, 2020). Initiatives such as the Reach Out and Read (ROR) program in the US demonstrate that interventions carried out by pediatricians during ordinary well-child consultations are effective in increasing shared reading and literacy-focused activities among parents of preschoolers (for a review, see Klass et al., 2009). Similar programs (*Nati per Leggere*; litt.: Born-to-Read) have also been implemented in Italy.^1^ The *Nati per Leggere* program, for instance, has helped the promotion of shared storybook reading and other literacy-focused activities at age 0–6 years, and has currently become routine advice during well-child consultations (Toffol et al., 2011).

Ordinary well-child visits may therefore be a valid setting for also presenting parents with guidance on developmentally appropriate activities to foster children’s emerging mathematical skills (Purpura et al., 2019). To the best of our knowledge, however, no systematic initiative has been taken to date to include guidance to home numeracy activities as a part of pediatric consultations to parents of preschool-aged children.

### The present study

The primary aim of the current work was to investigate whether parents’ involvement in a non-intensive intervention delivered by community pediatricians during scheduled well-child consultations at age 5 was associated with subsequent parents’ engagement in shared mathematics-related activities at home, as well as with children’s early numeracy development. To this end, the *Nati per Contare* (litt.: Born-to-Count) program was developed in cooperation between the local health authority of the district of XXXX, the Universities of YYYY and ZZZZ (blinded for review purposes), and an Italian professional association of pediatricians (Associazione Culturale Pediatri Romagna – ACPR). Community pediatricians involved in the Born-to-Count program were trained to provide parents with advice on the importance of early numerical competencies, and guidance on home mathematics-related activities that could be easily implemented in daily family routines (e.g., cooking activities, board games, shared reading of storybooks with numerical contents). The primary expected outcome was a steeper increase in the provision of mathematics-related activities, as compared to a baseline assessment conducted during the first preschool year, at age 3, among parents who received the Born-to-Count intervention, relative to those in a business-as-usual control condition who did not receive any numeracy-related advice during well-child consultations. The secondary expected outcome was an improvement in children’s performance on a standardized assessment of early numeracy from baseline to the end of the third preschool year. Feasibility and acceptability of the Born-to-Count intervention were also assessed.

## Materials and methods

### Participants and procedure

Participants were 204 parents of children (111 boys, 93 girls) attending to 11 public and private childcare centers. All the children were patients of 24 community pediatricians in the district of XXX, Italy, a local area that is characterized by generally favorable economic indicators (see: https://www.istat.it/storage/urbes2015/cesena.pdf). Attendance at scheduled well-child visits at 5 years in the district of XXX is 88% (Regione Emilia-Romagna, 2020).

Participants were part of a larger sample of parents and children involved in a multi-center longitudinal study on factors promoting early numerical development (*N* = 256) which was conducted in the district of XXX and in other districts in Northern Italy (see Authors, 2022, for details). Beyond focusing on early numerical development and parental provision of mathematics-related activities, the larger study also included measures that were not taken into consideration in the current research (e.g., parental provision of literacy-focused activities). Only participants resident in the district of XXX, where the Born-to-Count program was implemented, were recruited for the present study. Children’s diagnosis of neurodevelopmental disorders and parents’ being non-Italian speaking were criteria for exclusion.

**Figure 1:**
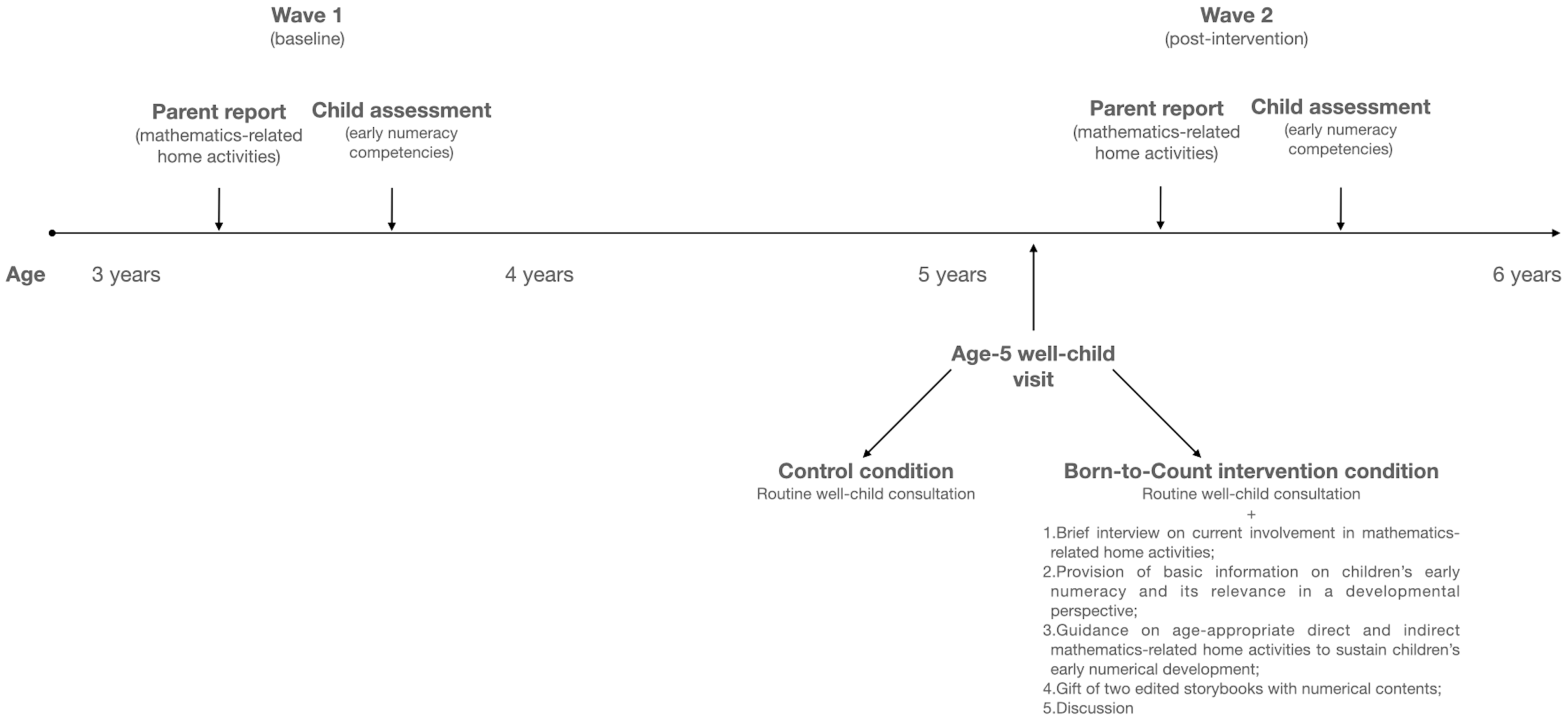
Timeline of data collection waves in the Born-to-Count study.

Recruitment took place through childcare centers when children were attending the first preschool year. Parents who provided informed consent to take part in the study were asked to complete a questionnaire to report their mathematics-related practices and other relevant study variables when children were in their first preschool year (Wave 1; children’s *M*_age_ = 45.78 months, *SD* = 3.21; range: 39–51 months). One-to-two months after scheduled attendance at the well-child visit at 5 years of age, parents were asked to complete the same questionnaire again (Wave 2). Parents choose whether to complete questionnaires in paper-and-pencil or in electronic format. The completion format was unrelated to the outcomes. Children’s assessments were conducted at the onset of the Study (Wave 1), and then repeated when children were attending the third and last preschool year (Wave 2; *M*_age_ = 67.75 months, *SD* = 3.17; range: 62–73 months). Wave 2 data collection with children occurred on average 6 months after the scheduled 5-year-old well-child visit. The study timeline is reported in Figure 1. Both parents and children were invited to participate in data collections at Wave 2 regardless of their participation in Wave 1.

One hundred and seventy-two parents and 195 children took part in the study at Wave 1, and 174 parents and 190 children participated at Wave 2. One hundred and sixty-one children and 150 parents participated in the study at both waves. Most of both mothers (*n* = 152, 74.5%) and fathers (*n* = 160, 78.4%) were born in Italy; 21 mothers (11.8%) and 13 fathers (6.4%) were born in other countries (predominantly Europe and Northern Africa). Information regarding nationality was missing for 31 mothers (15.2%) and 31 fathers (15.2%). As regards education levels, 21 mothers (10.3%) and 43 fathers (21.1%) had a middle school diploma or lower, 64 mothers (31.4%) and 70 fathers (34.3%) had a high school education, 88 mothers (43.1%) and 55 fathers (27.0%) had a bachelor’s degree or higher. Information regarding education levels was not provided for 31 mothers (15.2%) and 36 fathers (17.6%). Thus, most families were middle-class and both parents and children were born in Italy. As in most questionnaire studies, mothers responded to the questionnaire.

The study protocol was approved by Ethical Committee of the University of YYYY and by the Ethical Board of the Local Health Authority of WWWW.

### Intervention

Assignment of participants to the Born-to-Count intervention versus a business-as-usual control condition was determined at the pediatrician level (i.e., cluster randomization). Specifically, all parents whose children were patients of a community pediatrician selected to deliver the Born-to-Count were allocated to the intervention condition, whereas parents of children in charge to all other pediatricians were included in the control condition. The decision to follow a cluster randomization procedure was intended to avoid treatment disparities among patients of the same clinician. A person from the Local Health Authority who was not involved in the study divided community pediatricians into two groups in order to have approximately the same number of children in each group. One of the two groups (which included seven pediatricians) was then randomly assigned to the Born-to-Count intervention condition. The other 17 pediatricians were assigned to the control condition. Pediatricians in the control condition conducted regular well-child visits according to the standard protocol adopted by the Local health Authority in XXXX and received no instruction with regard to the promotion of mathematics-related home activities.

Pediatricians in the intervention condition received a 3-h training session led by three of the authors (CT, FC, and GB) to illustrate the purpose, procedures, and materials in Born-to-Count program. The intervention was designed as a single session to be delivered by the pediatrician to the parents at the routine well-child visit in the 5th year of age of the child, right after having completed all the scheduled assessments (e.g., growth patterns, dental health, eating habits). In detail, the Born-to-Count intervention protocol was designed as follows:

1. First, pediatricians were invited to briefly interview parents on children’s acquisition of emerging numeracy skills (e.g., “Did you notice whether your child uses fingers to count?”) and their current involvement in mathematics-related activities in the home environment (e.g., “Do you do any activity with your child that involves using numbers? For example, playing dice or card games? Or counting and measuring ingredients when cooking?”).
2. Then, pediatricians gave parents and discussed with them a printed booklet edited by the Local Health Authority. The booklet included:
  - basic information on children’s numerical development from 0 to 5 years (e.g., the acquisition of the number-word sequence, or the cardinality principle) and its relevance in a developmental perspective;
  - guidance on age-appropriate mathematics-related practices to sustain children’s early numerical development (e.g., involvement in daily activities that require measurement, counting, or doing simple sums);
  - suggestions on edited storybooks (e.g., *Inch by Inch* by Leo Lionni) that provide numeracy content (these were available at the local public library) and board games with developmentally-appropriate numerical content.

As guidance to shared home numeracy activities, the pediatricians were instructed to provide detailed examples of activities described in the above-mentioned booklet, pertaining to:

- direct mathematics-related activities, such as drawing attention to numerical symbols in the child’s environment (e.g., road signs, timetables), helping the child counting objects, and doing simple operations;
- indirect mathematics-related activities, including playing board games, or doing measurements during cooking activities;
- non-numerical activities that are related to numerical development, such as visuo-spatial activities (e.g., building blocks).

Finally, pediatricians gifted two storybooks with numerical contents to parents. Before concluding the well-child visit, pediatricians asked parents for any clarification or further information, if needed, and encouraged them to incorporate the mathematics-related activities into their daily home routines.

### Measures

#### Outcomes

##### Mathematics-related activities

The frequency of parent-reported mathematics-related activities was assessed through 20 items drawn from a widely used home numeracy questionnaire by Skwarchuk et al. (2014). Parents were asked to report how frequently they engaged in a list of activities (e.g., “I help my child to recite numbers in order,” “We play games that involve counting, adding, or subtracting,” or “My child adds and stirs ingredients that I measure”; see details in Table 1). Response scale ranged from 1 (never) to 5 (almost daily).

**Table 1:** Raw scores of parent-reported frequency of mathematics-related home activities and children’s early numeracy at Wave 1 (baseline) and Wave 2 (post-intervention).

	Wave 1 (baseline)		Wave 2 (post-intervention)	
	Mean	*SD*	Mean	*SD*
Mathematics-related activities				
We talk about time with clocks and calendars	2.49	1.474	2.89	1.408
I encourage my child to do math in his or her head	1.73	1.141	2.41	1.299
We sing counting songs (e.g., “Five Little Monkeys”)	3.07	1.389	2.77	1,327
We play games that involve counting, adding or subtracting	2.12	1.301	2.79	1.187
We time how fast an activity can be completed	1.69	1.152	2.20	1.289
I help my child to recite numbers in order	3.49	1.287	3.41	1.263
We play board games or cards	2.65	1.243	3.09	1.231
I ask about quantities (e.g., how many spoons?)	3.34	1.278	3.59	1.238
I encourage collecting (e.g., cards, stamps, rocks)	1.67	1.105	2.02	1.329
I encourage use of fingers to indicate ‘how many’	3.65	1.343	3.52	1.29
I help my child weigh, measure and compare quantities	2.18	1.257	2.40	1.166
I help my child learn simple sums (e.g., 2 + 2)	1.88	1.138	2.70	1.314
We discuss measurement terms (1/2 cup versus 1/4 cup)	1.90	0.882	2.28	0.933
My child adds and mixes what I measure	2.60	0.815	2.67	0.888
My child does most of the measuring, with some help	1.72	0.848	1.97	0.872
My child watches while I measure and stir ingredients	2.48	0.838	2.47	0.924
My child counts (with fingers, aloud) while we are cooking	1.89	0.885	2.2	0.93
My child weight the ingredients	1.56	0.747	1.97	0.935
My child divides or multiplies ingredients	1.12	0.378	1.23	0.540
My child compares quantities and says which ingredients are more present than others (notions “lesser than,” “greater than”)	1.75	0.874	1.98	0.955
My child can recognize different kinds of ingredients but with the same quantity (notion “as large as”)	1.53	0.756	1.81	0.884
Children’s early numeracy				
BAS-3	12.04	6.17	22.69	4.85

*N* = 204. BAS-3: British Ability Scales (Early Number Concepts sub-test). Range for parent-reported mathematics related activities: 1 (never) – 5 (almost daily). Range for BAS-3 scores at Wave 1: 0–28 (observed range: 0–26). Range for BAS-3 scores at Wave 2: 0–29 (observed range: 2–29). Higher BAS-3 scores indicate better performance.

##### Children’s early numeracy

The Early Number Concepts sub-test from the British Ability Scales (BAS-3; Elliot and Smith, 2011) was used to assess different aspects of children’s early numeracy (i.e., quantity understanding, number concepts, symbol-quantity mapping, counting, ordinality, cardinality, and simple arithmetic). One point is assigned for each correct answer, and testing terminates once a child produced five consecutive errors. Performance raw score is calculated as the sum of correct responses. The scale is validated for use with children between 3 and 7 years of age.

#### Feasibility and acceptability of intervention

Feasibility was assessed by collecting data from pediatricians and parents in the Born-to-Count intervention condition. Pediatricians in the Born-to-Count intervention group were individually interviewed to determine whether (a) the intervention was compatible with the timing of a regular well-child visit, and (b) parents reported positive or negative comments on the intervention. To evaluate acceptability, parents in the Born-to-Count intervention condition were asked to complete a supplementary section in the Wave 2 parents’ questionnaire with 11 items regarding satisfaction and enjoyment with the pediatrician’s advice (e.g., “The pediatrician’s recommendations were easy to implement”) and the contents of the Born-to-Count intervention booklet (e.g., “The Born-to-Count booklet was clearly written”), as well as the appropriateness of the received guidance for the child’s age and needs (e.g., “Activities suggested in the Born-to-Count booklet were too easy for my child’s age”). Response scale ranged from 1 (completely disagree) to 4 (completely agree).

### Data analyses

The software program IBM SPSS 27 was used to carry out analyses. Descriptive statistics are expressed as frequencies for categorical data, and as mean scores, standard deviation (*SD*), range (i.e., minimum and maximum observed scores), skewness, and kurtosis for all continuous outcomes. Single-group *t*-tests against the scale mid-point were used to analyze parents’ responses to items assessing feasibility and acceptability of the Born-to-Count intervention.

For the outcome measures, an intention-to-treat analytical approach was adopted, and linear mixed-effects (LME) models were used to assess change over time and group differences between participants in the Born-to-Count intervention and in the control condition for mathematics-related home activities and children’s early numeracy. LME models offer several advantages over traditional analytical approaches to longitudinal data analysis in intervention trials, especially in presence of unbalanced designs (i.e., with unequal number of participants within each level of a grouping variable), incomplete data (e.g., with missing observations at one time point), and non-independence among observations (e.g., with multiple observations for each participant, or with participants nested within contexts; Westfall et al., 2014). An additional advantage of LME models is that they handle each observation at a time point as a unit of analysis (instead of each individual participant), thus allowing to account for variability not only across participants, but also across indicators of the study constructs (i.e., survey items) over time.

In detail, we estimated two random-intercept LME models with mathematics-related home practices and children’s early numeracy, respectively, as the outcomes, and wave (within-participants: one and two), condition (between-participants: Born-to-Count intervention vs. control), and wave by condition interaction as the fixed factors. Two random intercept factors were also included in the LME models to account for participant-specific and pediatrician-specific variability in the outcome measures. In the case of the LME model on mathematics-related home practices, an additional random factor was included to account for item-specific variability. In presence of significant fixed interaction effects, post-hoc simple slope models were computed to detect specific trends over time in the outcome variable among participants in the Born-to-Count intervention and in the control condition, respectively, after accounting for participant-specific, pediatrician-specific, and item-specific random variability.

## Results

Descriptive statistics for study variables are reported in Table 1. Preliminary analyses revealed that participants in the control vs. Born-to Count intervention conditions did not differ at baseline (Wave 1) on any demographic characteristics or study measures (details are reported in Supplementary Table A1).

### Feasibility and acceptability of intervention

As regards feasibility, pediatricians in the intervention condition (*N* = 7) reported that the Born-to-Count intervention required on average 15 additional minutes relative to the usual duration of well-child visits at age 5. All the pediatricians also reported that the Born-to-Count intervention was fully compatible with the ordinary management of well-child visits. Five out of seven pediatricians reported that parents were apparently “very interested” in the contents of the Born-to-Count intervention, and two reported that parents were on average “quite interested.”

As regards parents, 90% of participants in the Born-to-Count intervention condition reported having received specific information and advice on children’s numerical development from the pediatrician. It is worth noting that 25% of parents in the control condition also reported having received advice on children’s early numeracy, even though numerical development is not included in the protocol of routine well-child visit at age 5. In addition, 79.3% of parents in the intervention condition reported having read the Born-to-Count booklet after the well-child visit.

As regards acceptability, between 88.3% and 98.5% of parents in the Born-to-Count intervention condition reported positive or very positive evaluations of the advice from the pediatrician and the contents of the informative booklet (e.g., interesting, easy to understand, helpful). Between 7.7% and 9.7% of parents in the Born-to-Count intervention condition reported that the Born-to-Count guidance was slightly or too difficult to implement. At the same time, 23.9% of parents rated the proposed activities as slightly or definitely too easy for the age of the child. Overall, single-group *t*-tests against the scale mid-point revealed that all positively-worded items displayed average scores that were significantly above the scale mid-points (all *t*_(0)_s > 10.420, all *p*s < 0.001), thus indicating a general appreciation of the pediatrician’s advice in support of home mathematics-related activities. Similarly, all negatively worded items displayed average scores that were significantly below the scale mid-point (all *t*_(0)_s > 6.258, all *p*s < 0.001), indicating that the pediatrician’s advice and the suggested mathematics-related activities were mostly deemed as appropriate to the children’s age and developmental needs. Details are reported in supplemental materials (Supplementary Table A2).

### Outcomes

#### Mathematics-related activities

Results from the LME model for the parents’ reports of mathematics-related activities are presented in Table 2.

**Table 2:** Estimates from Linear Mixed Effects (LME) models on parent-reported frequency of mathematics-related home activities and children’s early numeracy.

	Mathematics-related home activities		Children’s early numeracy	
	Estimate (*SE*)	Value of *p*	Estimate (*SE*)	Value of *p*
Fixed components				
Intercept	2.333 (0.143)	<0.001*	17.517 (0.422)	<0.001*
Wave	0.140 (0.011)	<0.001*	5.388 (0.237)	<0.001*
Condition	0.001 (0.037)	0.991	0.672 (0.422)	0.140
Wave * Condition	0.029 (0.013)	0.023*	0.123 (0.237)	0.605
Random components				
Participant	0.183		9.717	
Item	0.401		–	
Pediatrician	<0.001		0.894	
Residual	1.050		19.572	

*N* = 204. Estimates for random components represent variances (σ2). *p < 0.05.

**Figure 2:**
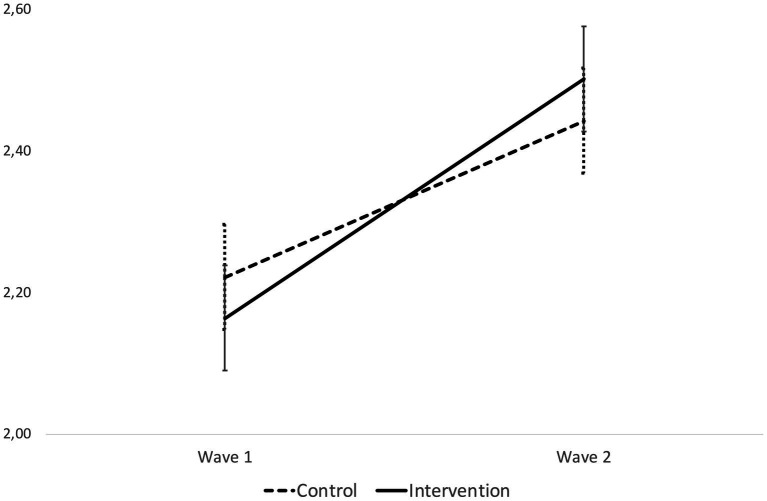
Trends in frequency of home mathematics-related activities from baseline to post-intervention for participants in the Born-to-Count intervention versus control condition. Error bars represent Standard Errors.

Estimates for the fixed components of the model reveal that the main effect of wave was significant, thus indicating that the overall frequency of mathematics-related home activities increased significantly from Wave 1 to Wave 2. The main effect of condition was not significant. However, a significant interaction between condition and wave emerged (*β* = 0.029, *SE* = 0.013, *p* = 0.023), indicating – as predicted – that change in the frequency of reported mathematics-related activities from baseline to post-intervention assessment was different in size between participants in the intervention and those in the control condition. Estimated trends in frequency of mathematics-related activities over time are depicted in Figure 2.

As it is evident from Figure 2, the difference in point estimates for the frequency of mathematics-related home activities between participants in the control and in the intervention condition was not significant neither at Wave 1 (*M*_control_ = 2.222, *SE*_control_ = 0.150; *M*_intervention_ = 2.164, *SE*_intervention_ = 0.148; *F*_(1,197)_ = 0.550, *p* = 0.459) nor at Wave 2 (*M*_control_ = 2.443, *SE*_control_ = 0.150; *M*_intervention_ = 2.502, *SE*_intervention_ = 0.147; *F*_(1,195)_ = 0.586, *p* = 0.446). However, the slope representing the increase in the frequency of mathematics-related home activities between Wave 1 and Wave 2 was significantly steeper for participants exposed to the Born-to-Count intervention (*β* = 0.169, *SE* = 0.17, *p* < 0.001), as compared to participants in the control condition (*β* = 0.111, *SE* = 0.19, *p* < 0.001).

Estimates for the random part of the model reveal that variability across participants and variability across items represent approximately the 26% and the 11%, respectively, of the observed variability that is not accounted for by the fixed part of the model, whereas variability due to participants’ nesting within pediatricians is close to zero.

#### Children’s early numeracy

Results from the LME model for the analysis of children’s early numeracy are presented in Table 2. A significant main effect of wave emerged, indicating that children’s early numeracy improved from Wave 1 to Wave 2, as expected. In contrast, neither the main effect of condition nor the wave by condition fixed effects were significant, thus indicating that parents’ involvement in the Born-to-Count intervention did not produce differential changes over time in children’s early numeracy compared to the control condition.^2^

In the random part of the model, estimates reveal that variability across participants amounts to ~32% of the variability that is not accounted for by the model’s predictors, whereas variability due to participants’ nesting within pediatricians is close to zero (0.03%).

## Discussion

The preschool years are a critical age period for the acquisition of foundational skills and prerequisites for subsequent children’s mathematical development (LeFevre et al., 2010a; Watts et al., 2014). Parents’ provision of shared mathematics-related activities in the home environment is associated with the growth of children’s mathematical skills prior to formal schooling (Mutaf Yıldız et al., 2020). The goal of the present study was to examine whether a non-intensive intervention delivered by community pediatricians in the context of ordinary well-child visits would increase the frequency of mathematics-related activities at home and promote the growth of children’s early numeracy over the preschool years. Specifically, pediatricians working in community health services were trained to deliver information to parents concerning the emergence and the importance of children’s early numerical skills prior to formal schooling, and to provide guidance on developmentally-appropriate shared activities in the home environment that may sustain early numerical development. The contents and the format of the materials developed for the Born-to-Count intervention on mathematics-related home activities were modeled on an existing program used to promote shared literacy-focused activities in the home environment (i.e., the *Nati per Leggere* program; https://www.natiperleggere.it), which is currently a routine protocol of pediatric well-child visits in Italy.

Overall, community pediatricians involved in the study reported that the intervention was feasible and sustainable. Providing advice on numerical development and mathematics-related activities was deemed as highly compatible with the overall context of well-child consultations, in which guidance is routinely provided to parents over several other aspects of child development (e.g., nutrition, physical activity, dental health, early literacy). The Born-to-Count intervention was also deemed as highly acceptable by parents, who reported high levels of satisfaction and enjoyment with the guidance provided by the pediatricians, and generally rated the suggested mathematics-related activities as appropriate to their child’s age and developmental needs. In sum, pediatricians’ and parents’ feedback suggests that routine well-child consultations may represent a valid setting for promoting activities to foster children’s early mathematical skills (Mazzocco, 2016; Purpura et al., 2019).

Consistent with expectations, parents who received the Born-to-Count intervention reported an increased frequency of mathematics-related activities in the home environment at the post-intervention assessment – relative to baseline – to a greater extent than parents in the control condition, who received a business-as-usual well-child visit. These findings are consistent with those of other intervention studies (Starkey and Klein, 2000; Berkowitz et al., 2015; Niklas et al., 2016; Leyva et al., 2018). However, in most cases, previous studies used more intensive interventions (e.g., repeated encounters with parents over prolonged time periods). Intensive interventions may be more powerful, but they may also limit participation and feasibility, due to features such as self-selection of participants, effort required, and attrition over the course of the intervention. In contrast, the current research adds to the few existing studies showing that even non-intensive interventions that involve minimal engagement from families, can result in positive outcomes, and contribute to the inclusion of mathematics-related activities in the home environment. Moreover, the current intervention can be provided within the context of well-child visits that families would attend anyway. Accordingly, this intervention has no additional cost to the parents. In health systems in which access to primary care is universal and free-of-charge for the whole population, as in Italy (Corsello et al., 2016), well-child consultations administered by community pediatricians are a context in which sensitivity to the importance of children’s numerical development, and its crucial impact later in life, can be promoted to all families.

Despite showing that the intervention was successful in influencing parents’ reports of the frequency of mathematics-related home activities, the Born-to-Count intervention did not have an impact on the skills of children whose parents received guidance from pediatricians, relative to those who did not. There was indeed a substantial increase in early numeracy skills for all children between 3 and 5 years of age, but the increase was not significantly different for participants in the intervention condition compared to those in the control condition. In part, these findings confirm the difficulty of influencing children’s competencies through parent-based training programs. Meta-analytic findings suggest that the size of positive effects of parent-based interventions that promote mathematics-related competencies in the preschool years are quite small (Cohen’s *d* = 0.18; Cahoon et al., 2022).

In the present study, one potential explanation for the null effect of the Born-to-Count intervention on the children’s early numeracy is the low intensity of the intervention, which consisted of about 15 min of discussion and delivery of informative materials within the context of a well-child visit. Whereas the low intensity of the intervention supports feasibility and sustainability, it may also limit the potential long-term impact of the intervention itself. Moreover, parental report of mathematics-related home activities occurred only 2–4 months before the assessment of children’s’ numeracy in the post-intervention phase. We speculate that changes in the parent-reported frequency of mathematics-related activities at home may require more time to reflect in benefits to children’s early numeracy. Future research is needed that involves monitoring both parents’ mathematics-related practices and children’s early numeracy over a more prolonged time span.

### Limitations and future directions

The present research is one of the few studies testing the impact of an intervention targeted to parents that was designed to promote mathematics-related activities in the home environment in the preschool years. Moreover, as far as we know, it is the very first study that relied on community pediatricians as a resource to promote the development of children’s mathematical skills. Nevertheless, this study has several limitations. First, parents’ mathematics-related home activities were indexed through a self-report measure. Although this measure has been widely used in many previous studies in the field (Skwarchuk et al., 2014), and is consistently related to standardized measures of children’s numerical competencies (e.g., Napoli and Purpura, 2018; Purpura et al., 2020; Susperreguy et al., 2021), parents’ reports may be biased by social desirability concerns. Although more time- and resource-consuming, future studies may benefit from integrating parent-reported measures with observation-based assessment of shared mathematics-related activities.

Second, only two measurement waves were included in the present study. The inclusion of repeated waves of assessment both at the pre- and at the post-intervention phase would allow a more fine-grained modeling of individual trajectories of change over time in the outcome measures and would also allow a considerable increase in statistical power (Toffalini et al., 2021). Moreover, the assessment of children’s numeracy skills when children enter school and first encounter formal teaching of mathematics, would provide a stronger test of the persistence of the intervention outcomes. Similarly, delivering the intervention at earlier ages (e.g., in the first or second preschool year) may be important, as this would allow more time for parents to include mathematics-related practices in their home routine before children enter primary school. Longer-term follow up is also important considering the accumulating evidence of fade-out effects for numerous early childhood education programs (Abenavoli, 2019).

Third, the strength of the intervention may have been insufficient. It included several components, such as interviewing of parents concerning their current mathematics-related practices, information on children’s early numerical development, guidance on diverse mathematics-related activities to be incorporated in family routines, and delivery of printed materials and storybooks with numerical contents. Although all these elements are associated with positive outcomes in previous intervention studies in the field, the design of the current study does not allow us to disentangle which of these elements affected parents’ provision of mathematics-related activities. Because routine well-child visits have time constraints, and parents are simultaneously provided with information regarding several aspects of the child health and development at these visits, focusing attention on only a few critical and most impactful elements may help increase the effectiveness of pediatricians’ guidance.

Finally, the frequency of related but non-numerical home activities was not assessed. Parents in the Born-to-Count intervention conditions were also encouraged to engage in practices that may indirectly foster children’s numeracy skills, such as visuo-spatial activities and shared reading of storybooks with numerical content. Furthermore, it may be important in future studies to also monitor the possible impact of interventions to promote mathematics-related activities not only on children’s numeracy skills, but also on their emerging self-concept in mathematics, or on emotions toward mathematics (e.g., math anxiety).

## Conclusion

In conclusion, we found that a non-intensive intervention implemented within the context of routine well-child visits at age 5 was associated with a larger increase in the frequency of parent-reported mathematics-related activities in the home environment, compared to parents who received an ordinary well-child consultation. These findings add to the limited body of research on interventions to promote mathematics-related activities in the home environment in the preschool years and identify, for the first time, community pediatricians and the public primary health care services as an important resource to support parents’ engagement in children’s early mathematical development.

## Data availability statement

The original contributions presented in the study are included in the article/Supplementary material, further inquiries can be directed to the corresponding author.

## Ethics statement

The studies involving human participants were reviewed and approved by Bioethics Committee of the University of Bologna, Bologna, Italy, and Ethics Committee IRST and Area Vasta Romagna, Meldola (FC), Italy. Written informed consent to participate in this study was provided by the participants’ legal guardian/next of kin.

## Author contributions

CT wrote the initial draft of the manuscript and ran the statistical analyses. J-AL, MP, CV, and VG contributed to different versions of the manuscript and were involved in the revision and interpretation of the results. VG contributed to the data collection process and the raw data preparation. CT, J-AL, MP, AB, FC, and GB contributed to the design of the study and the “Born-to-Count” intervention protocol. AB, FC, and GB obtained funding to carry out the study. All authors agreed to all aspects of the study, and approved the final version of the manuscript.

## Funding

The study was partly funded through a contribution awarded by Fondazione Cassa di Risparmio di Cesena to Associazione Culturale Pediatri – Romagna (ACPR). The funder was not involved in the study design, collection, analysis, interpretation of data, the writing of this article, or the decision to submit it for publication.

## Conflict of interest

The authors declare that the research was conducted in the absence of any commercial or financial relationships that could be construed as a potential conflict of interest.

## Publisher’s note

All claims expressed in this article are solely those of the authors and do not necessarily represent those of their affiliated organizations, or those of the publisher, the editors and the reviewers. Any product that may be evaluated in this article, or claim that may be made by its manufacturer, is not guaranteed or endorsed by the publisher.

## Footnotes

^1^ https://www.natiperleggere.it

^2^A supplemental analysis was also performed using Generalized Estimation Equation (GEE) in order to determine whether the intervention produced differential outcomes depending on baseline levels of parents’ provision of mathematics-related activities or children’s early numeracy skills at Wave 1. No interaction emerged between condition (control vs. Born-to-Count intervention) and baseline levels of the outcome variables (both *p*s > 0.800).
