# Supplementary material for: With a little help from our pediatrician: An intervention to promote mathematics-related home activities through regular well-child visits
Source: Front Psychol. 2022 Dec 5;13:1051822. doi: 10.3389/fpsyg.2022.1051822 (PMC9763055; doi:10.3389/fpsyg.2022.1051822)
Supplement: Supplementary file 1 [file Data_Sheet_1.pdf]

Supplementary Material

1 Supplementary Table A1

Comparison of demographic information and study variables between participants in the control vs. Born-to-Count intervention condition at baseline (Wave 1; Children: N = 174; Parents: N = 172).

	Control condition		Born-to-Count intervention condition		Test statistics	*p* value
	n or mean	% or *SD*	*n* or mean	% or *SD*	*Chi*^{2} or *t*	
Demographics						
Children's age (months)	45.39	3.12	45.86	3.25	0.539	0.556
Children's sex						
Female	38	43.7%	56	51.9%	1.289	0.256
Male	49	56.3%	52	48.1%		
Mothers' education						
Middle school or lower	6	7.8%	15	15.6%	2.591	0.274
High school	31	40.3%	33	34.3%		
Bachelor's degree or higher	40	51.9%	48	50.0%		
Fathers' education						
Middle school or lower	16	21.1%	27	29.3%	1.839	0.399
High school	32	42.1%	38	41.7%		
Bachelor's degree or higher	28	36.8%	27	29.3%		
Mothers' country of birth						
Italy	66	86.8%	86	88.7%	0.132	0.716
Other	10	13.2%	11	11.3%		
Fathers' country of birth						
Italy	70	92.1%	90	92.8%	0.028	0.867
Other	6	7.9%	7	7.2%		
Mathematics-related activities						
We talk about time with clocks and calendars	2.51	1.54	2.4	1.47	0.411	0.681
I encourage my child to do math in his or her head	1.71	1.16	1.76	1.16	0.245	0.806
We sing counting songs (e.g., "Five Little Monkeys")	2.91	1.39	3.08	1.38	0.757	0.450
We play games that involve counting, adding or subtracting	2.01	1.25	2.16	1.31	0.707	0.481

We time how fast an activity can be completed	1.64	1.16	1.66	1.15	0.111	0.912
I help my child to recite numbers in order	3.43	1.22	3.53	1.35	0.483	0.630
We play board games or cards	2.82	1.12	2.48	1.29	1.652	0.101
I ask about quantities (e.g., how many spoons?)	3.49	1.25	3.17	1.29	1.544	0.125
I encourage collecting (e.g., cards, stamps, rocks)	1.61	1.07	1.58	1.02	0.167	0.867
I encourage use of fingers to indicate 'how many'	3.81	1.23	3.55	1.41	1.187	0.237
I help my child weigh, measure and compare quantities	2.30	1.29	1.96	1.15	1.684	0.094
I help my child learn simple sums (e.g., 2 + 2)	1.88	1.14	1.93	1.17	0.263	0.793
We discuss measurement terms (1/2 cup versus 1/4 cup)	2.02	0.87	1.79	0.89	1.503	0.135
My child adds and mixes what I measure	2.60	0.81	2.48	0.84	0.825	0.411
My child does most of the measuring, with some help	1.68	0.88	1.73	0.87	0.392	0.695
My child watches while I measure and stir ingredients	2.40	0.93	2.49	0.80	0.667	0.506
My child counts (with fingers, aloud) while we are cooking	1.86	0.86	1.87	0.90	0.037	0.970
My child weight the ingredients	1.57	0.71	1.53	0.79	0.314	0.754
My child divides or multiplies ingredients	1.14	0.39	1.12	0.36	0.282	0.778
My child compares quantities and says which ingredients are more present than others (notions "lesser than", "greater than")	1.75	0.88	1.75	0.90	0.027	0.979
My child can recognize different kinds of ingredients but with the same quantity (notion "as large as")	1.54	0.75	1.52	0.74	0.119	0.905
Children's early numeracy						

BAS-3	13.00	6.13	11.17	6.16	1.958	0.052

Note. BAS-3: British Ability Scales (Early Number Concepts sub-test). Range for parent-reported mathematics related activities: 1 (never) – 5 (almost daily). Range for BAS-3 scores: 0-26 28 (observed range: 0-26). Higher BAS-3 scores indicate better performance.

2 Supplementary Table A2

Raw scores of parent-reported ratings of feasibility and acceptability of the Born-to-Count intervention (N = 98).

Measure	Descriptives		Single-group t-tests against scale mid-point (2.5)		
			$t_{(0)}$	*p*	Cohen's *d*
	M	*SD*			
The pediatrician's recommendations were helpful	3.43	0.630	14.413	<.001	1.479
The pediatrician's recommendations were easy to implement	3.32	0.511	15.566	<.001	1.597
Activities suggested by the pediatrician were too difficult for my child's age	1.53	0.731	-12.835	<.001	1.331
Activities suggested by the pediatrician were too easy for my child's age	1.83	0.867	-7.538	<.001	0.769
The Born-to-Count booklet was clearly written	3.42	0.721	10.423	<.001	1.273
The Born-to-Count booklet was difficult to understand	1.34	0.668	-14.020	<.001	1.739
The Born-to-Count booklet was too long	1.45	0.788	-10.778	<.001	1.327
The Born-to-Count booklet was interesting	3.58	0.583	14.988	<.001	1.859
The Born-to-Count booklet was helpful	3.48	0.756	10.420	<.001	1.302
Activities suggested in the Born-to-Count booklet were too difficult for my child's age	1.68	0.768	-8.657	<.001	1.066
Activities suggested in the Born-to-Count booklet were too easy for my child's age	1.89	0.779	-6.258	<.001	0.782

Note. Range for parent-reported mathematics feasibility and acceptability rating of Born-to-Count intervention: 1 (completely disagree) to 4 (completely agree).
